# Supragingival plaque microbial analysis in reflection to caries experience

**DOI:** 10.1186/1472-6831-13-5

**Published:** 2013-01-08

**Authors:** Alaa Mannaa, Anette Carlén, Guglielmo Campus, Peter Lingström

**Affiliations:** 1 Department of Cariology, Institute of Odontology, The Sahlgrenska Academy, University of Gothenburg, Box 450, Gothenburg, SE-405 30, Sweden; 2 Department of Conservative Dental Sciences, Faculty of Dentistry, King Abdulaziz University, P.O Box 80209, Jeddah, 21589, Kingdom of Saudi Arabia; 3 Department of Oral Microbiology and Immunology, Institute of Odontology, The Sahlgrenska Academy, University of Gothenburg, Box 450, Gothenburg, SE-405 30, Sweden; 4 Dental Institute, University of Sassari, Viale San Pietro 43/C, Sassari, I-07100, Italy; 5 WHO Collaborating Centre of Milan for Epidemiology and Community Dentistry, Via Beldiletto 1/3, Milan, I-20142, Italy

## Abstract

**Background:**

Dental caries develops as a result of the metabolism of carbohydrates by cariogenic bacteria present in a complex biofilm. The present study aimed to examine if bacteria in pooled supragingival plaque samples quantified using a “checkerboard DNA-DNA hybridization” based panel of caries-related bacteria, could reflect the caries experience in a manner similar to saliva samples analysed using a chair-side method in a previous investigation.

**Methods:**

A total of 86 mothers and their children aged 4–6 years and 12–16 years old participated. Caries experience (DMFT/dmft; Decayed, Missing and Filled Teeth for permanent and primary teeth) was registered clinically and radiographically. Caries was recorded at the D_3_ level (caries into dentine). The D/d component was divided into three categories. A pooled supragingival plaque sample per participant was obtained from posterior approximal sites. Analyses of 15 bacterial species were performed using the checkerboard DNA-DNA hybridisation technique.

**Results:**

No significant relationships were found between the bacterial scores and DMFT/dmft nor D/d groups.

**Conclusions:**

Unlike the saliva samples and the chair-side method, interproximal pooled plaque samples analysed using the “checkerboard DNA-DNA hybridization technique” did not reveal any significant relations between the bacterial counts and the caries experience.

## Background

Dental caries is a multifactorial disease initiated and influenced by a number of key factors [1]. In addition, saliva plays an important role in maintaining the teeth integrity by buffering acids produced by cariogenic bacteria and protecting teeth from decay. Saliva may influence the oral microflora by adsorbing to the tooth surface forming the acquired pellicle, which determines which microorganisms are able to attach and colonize [2]. Saliva has been conventionally used as a diagnostic tool to determine individual caries activity and risk [3,4]. Such assessment includes the recording of salivary buffer capacity and counts of cariogenic bacteria mainly mutans streptococci and lactobacilli using chair-side tests.

Although salivary analysis may provide a general overview of the oral ecology reflecting the caries risk, dental caries is principally a biofilm-induced disease [5]. Viewing this biofilm (dental plaque) as a complex microbial ecosystem has enhanced the understanding of its role in caries development and progression [6]. Cariogenic plaques result when acidogenic and aciduric bacterial species increase following high frequency carbohydrate exposure. The microbial metabolism of such carbohydrates will result in the acidification of the biofilm, which in turn may lead to acid-induced demineralization of the dental hard tissues [6]. One of the molecular techniques that have demonstrated the diverse microbial composition of the dental biofilm is the “checkerboard DNA-DNA hybridisation technique” [7,8]. However, no caries-specific panel has yet been proposed. It would therefore be interesting to use the checkerboard DNA-DNA hybridisation technique with a caries specific panel to investigate the relationship between individual caries experience and the counts of different caries-related supragingival plaque bacteria.

The present study was performed as part of a series of studies on Saudi mothers and their children. One part aimed at describing the caries experience in relation to various caries-related factors and a clear association between chair-side salivary bacterial counts and caries experience was found [9]. Another part was designed to determine if pooled interproximal plaque samples analysed using the “checkerboard DNA-DNA hybridisation technique” could reflect the caries experience.

The hypothesis of this cross-sectional study is that higher caries experiences are associated with higher counts of caries-related bacteria in supragingival plaque. The aim of the present study was therefore to examine if bacteria in pooled supragingival plaque samples quantified using a “checkerboard DNA-DNA hybridization” based panel of caries-related bacteria, could reflect the caries experience in a manner similar to saliva samples analysed using a chair-side method in a previous investigation.

## Methods

### Subjects

The study was given ethical approval by the Faculty of Dentistry at King Abdulaziz University in Jeddah, Kingdom of Saudi Arabia. The dental records at the Dental Health and Emergency Clinics at the Faculty of Dentistry were screened to identify suitable family candidates. The study comprised 86 volunteer families. From each family, the mother (mean age 37.0 ± 4.5 years) and two of her children, aged 4–6 years old (mean age 5.0 ± 0.9 years) and 12–16 years old (mean age 13.0 ± 1.4 years) volunteered. Verbal information about the study was given to the parents and written informed consent was obtained. All the investigations were carried out by one of the authors (AM).

### Caries registration

The dentition status was expressed using the DMFT index (Decayed, Missing and Filled Teeth) for permanent teeth and dmft for primary teeth. Third molars were excluded. Caries was registered clinically and radiographically at the D_3_ level [10]. Bitewing radiographs were taken for each participant in order to record approximal decay. All the approximal surfaces in the dentition from the mesial surface of the first premolars/primary molars to the distal surface of the second molars/primary molars were included.

### Bacterial plaque samples

Pooled supragingival plaque was sampled from the approximal sites between teeth 16/15, 25/26, 35/36 and 46/45 in the mothers and older children and teeth 55/54, 64/65, 74/75 and 85/84 in the younger children using sterile Gracey curettes. If one of the teeth in the approximal site was missing, an adjacent site was used. Each plaque sample was placed in an Eppendorf tube containing 150 *μ*l of sterile TE buffer (10 mM Tris–HCl, 1 mM EDTA, pH 7.6). Then 100 *μ*l of 0.5 M NaOH was added to the plaque pellet and the bacterial suspension was stored at -20°C pending further processing [11].

### Checkerboard DNA-DNA hybridisation

The analysis of bacterial species was performed using the checkerboard DNA-DNA hybridisation method [12]. Whole genomic probes were prepared from the 15 bacterial strains known to be related to caries as shown in Table 1. An evaluation of the bacterial count in the samples was performed by comparing the obtained signals with the ones generated by the pooled standard samples containing a count of 10^6^ and 10^5^ of each bacterial species, respectively. The signals were coded on a scale from 0 to 5 as follows: 0 = no signal; 1 = a signal density weaker than that of the low standard (<10^5^ bacteria); 2 = a signal density equal to that of the low standard (=10^5^ bacteria); 3 = a signal density higher than that of the low standard but lower than that of the high standard (>10^5^ but <10^6^ bacteria); 4 = a signal density equal to that of the high standard (=10^6^ bacteria) and 5 = a signal density higher than that of the high standard (>10^6^ bacteria). Further details regarding the technique are found in Mannaa et al. [13]. All the assessments were performed by one examiner (AM).

**Table 1:** The 15 caries-related bacterial strains used for the preparation of DNA probes and further comparison with clinical caries data

**Bacterial nomenclature**	**Strain description & source**
Mutans streptococci	
*Streptococcus mutans*	ATCC-25175
*Streptococcus sobrinus*	CCUG-27507
Non-mutans streptococci	
*Streptococcus mitis*	ATCC-49456D-5
*Streptococcus gordonii*	ATCC-35105D-5
*Streptococcus sanguinis*	ATCC-10556D-5
*Streptococcus salivarius*	ATCC-9759D-5
Lactobacilli	
*Lactobacillus casei*	ATCC-334D-5
*Lactobacillus salivarius*	CCUG-55845
*Lactobacillus fermentum*	OMGS-3182
Actinomyces	
*Actinomyces odontolyticus*	NCTC-9935
*Actinomyces oris*	ATCC-12104D-5
*Veillonella parvula*	ATCC-10790D-5
*Rothia dentocariosa*	CCUG-17835
*Bifidobacterium dentium*	OMGS-1956
*Parvimonas micra*	ATCC-33270

### Data analysis

All data were analysed using the IBM^®^ SPSS^®^ statistical package (PASW version 18.0, IBM^®^, Chicago, Ill., USA). The D/d component was categorised into low: ≤ 1, medium: 2–5 and high: > 5 for the mothers and older children, and low: ≤ 3, medium: 4–9 and high: ≥ 10 for the younger children. Cohen’s Kappa for the determination of the intra-examiner reliability of the caries registration was calculated. Descriptive statistics, including the means and standard deviations were calculated separately for the three groups. Regarding the checkerboard scores, only a few signal “0” data were recorded and therefore combined with signal “1” data into “≤1”. One-way ANOVA was used to compare mean differences in the caries experience in relation to the bacterial scores. Fisher’s exact test was used to associate the D/d groups with the bacterial scores. The level of statistical significance was set at 5%.

## Results and discussion

The Cohen’s kappa values for the caries registration for the mothers, younger children and older children were 0.88, 0.90 and 0.82 respectively. Table 2 summarizes the bacterial counts for each species. The caries experiences (DMFT/dmft) of the mothers, younger and older children are summarised in Table 3. 

**Table 2:** **Score distribution for the 15 bacterial strains obtained by the Checkerboard DNA-DNA Hybridization technique in the mothers, 4- to 6-year-old (C**_**4-6**_**) and 12- to 16-year-old (C**_**12-16**_**) children**

**Bacterial Strains**	**Checkerboard scores**														
	**Mothers**					**C**_**4-6**_					**C**_**2–16**_				
	**n = 86**					**n = 86**					**n = 86**				
	**≤1**	**2**	**3**	**4**	**5**	**≤1**	**2**	**3**	**4**	**5**	**≤1**	**2**	**3**	**4**	**5**
***S. mutans***	77	8	1	0	0	68	15	3	0	0	10	76	0	0	0
***S. sobrinus***	6	80	0	0	0	79	3	4	0	0	9	77	0	0	0
***S. sanguinis***	17	69	0	0	0	19	66	1	0	0	68	17	1	0	0
***S. salivarius***	84	2	0	0	0	80	6	0	0	0	83	3	0	0	0
***S. gordonii***	67	16	3	0	0	67	16	3	0	0	66	16	3	1	0
***S. mitis***	72	12	2	0	0	72	10	4	0	0	74	12	0	0	0
***L. casei***	81	5	0	0	0	82	4	0	0	0	81	5	0	0	0
***L. fermentum***	84	2	0	0	0	82	4	0	0	0	84	2	0	0	0
***L. salivarius***	84	2	0	0	0	84	2	0	0	0	83	3	0	0	0
***A. odontolyticus***	83	3	0	0	0	80	6	0	0	0	81	5	0	0	0
***A. oris***	55	27	4	0	0	40	40	6	0	0	55	30	1	0	0
***V. parvula***	56	28	1	1	0	50	29	7	0	0	54	29	3	0	0
***R.dentocariosa***	74	12	0	0	0	72	14	0	0	0	71	14	1	0	0
***B. dentium***	81	5	0	0	0	81	5	0	0	0	83	3	0	0	0
***P. micra***	20	28	24	14	0	24	26	28	8	0	29	19	29	8	1

**Table 3:** **Dentition status (DMFT/dmft) and caries experience (D/d) in the mothers, 4- to 6-year-old (C**_**4-6**_**) and 12- to 16-year-old (C**_**12-16**_**) children**

**Variable**	**Mothers**	**C**_**4-6**_	**C**_**12-16**_
	**n = 86**	**n = 86**	**n = 86**
DMFT/deft (mean ± SD)	12.4 ± 5.3	9.0 ± 5.0	5.8 ± 4.1
D/d (mean ± SD)	5.5 ± 3.9	8.0 ± 5.1	4.5 ± 3.7
DMFT/deft = 0 (n[%])	3[3.5]	8 [9.3]	10 [11.6]
D/d = 0 (n[%])	10 [11.6]	9 [10.5]	12 [14.0]
Low	17 [19.8]	23 [26.7]	23 [26.7]
Medium	31 [36.0]	25 [29.1]	32 [37.2]
High	38 [44.2]	38 [44.2]	31 [36.0]

There was an un-equal distribution of subjects within the scores on the checkerboard scale, with the majority of bacterial counts in the lower ranges of the scale. The analysis showed no relation between the bacterial scores and the caries experience for any of the caries-related bacteria. In addition, no significant associations were detected between the bacterial scores and the D/d categories in the mothers or in their younger or older children except for a tendency observed in relation to *Streptococcus mutans* as shown in Table 4.

**Table 4:** **Distribution of D/d in relation to *S. mutans *scores in the mothers, 4- to 6-year-old (C**_**4-6**_**) and 12- to 16-year-old (C**_**12-16**_**) children**

***S. mutans count***	**D/d**								
	**Mothers**			**C**_**4-6**_			**C**_**12-16**_		
	**Low**	**Medium**	**High**	**Low**	**Medium**	**High**	**Low**	**Medium**	**High**
	**(≤1)**	**(2–5)**	**(>5)**	**(≤3)**	**(4–9)**	**(>9)**	**(≤1)**	**(2–5)**	**(>5)**
1	17	29	31	20	18	30	23	30	27
2	0	2	6	3	4	8	0	2	4
3	0	0	1	0	3	0	-	-	-

The majority of studies using the checkerboard DNA-DNA hybridisation technique have focused on studying plaque bacterial ecology in relation to periodontal disease [14-17]. These studies revealed a directly proportional relationship between the bacterial count and periodontal pocket depth. The present study could not demonstrate a relationship between the counts of supragingival bacteria and the caries experience (DMFT/dmft). A relationship between caries experience and caries-related salivary bacterial counts (mutans streptococci and lactobacilli) assessed using CRT Bacteria^®^ was found in the same study sample [9]. In the present study, no relationship between the bacteria quantified in the pooled plaque samples and the caries experience was found. This corresponds well with an earlier study where a stronger association between dental caries and cariogenic organisms analysed by culture technique in comparison to the checkerboard method was found [18]. A number of factors related to the characteristics of dental plaque, the caries disease process and the choice of sampling method, may explain the current finding. The bacterial interaction in dental plaque could make a greater contribution to caries development than the specific count of certain cariogenic species. In addition, the plaque microbial community differs at different stages of plaque maturation (early plaque *versus* mature plaque). Furthermore, the location of the sampling sites and number of sites selected may influence the relationship. The count of plaque bacteria could also be affected by a number of caries-related factors other than the actual caries experience such as the location/depth (enamel *versus* dentine), stage (incipient *versus* cavitated), level (acute, chronic or arrested), and activity of the carious lesion (active *versus* inactive). Furthermore, the checkerboard DNA-DNA hybridisation technique has a high cut-off limit for bacterial quantification making the detection of bacteria in low counts problematic, which may be further complicated by cross-reactions [19]. Finally, the unequal distribution of subjects within the checkerboard scores with a majority of low bacterial counts in the present study could account for the lack of significant results.

In addition to the previously mentioned drawbacks, there are two limitations of the sampling method used in the present study. Unlike with saliva, it is difficult to standardize the amount of bacteria in a plaque sample. Standardization can be easily achieved with saliva by expressing the amount of bacteria in a 1-mL sample. Furthermore, pooling supragingival plaque into one test tube may create difficulties in lysing all bacterial cells present in a very dense bacterial suspension [20]. For this reason, heavy plaque sampling was minimized in the present study by taking only a sufficiently visible amount of supragingival plaque. However, the question whether too little plaque was collected cannot be ruled out.

Contrary to the present study, Aas et al. showed that higher counts of specific bacterial species were associated with health, caries initiation, and caries progression by using a reverse-capture essay [21]. Moreover, the present study analysed pooled plaque samples as opposed to samples from different caries-susceptible sites in the oral cavity. In addition, the DMFT/dmft index could be regarded as a crude method to describe the individual caries experience and conveys no information about caries activity. A relationship between plaque bacterial counts and caries experience may have been obtained if the investigation had been performed on a site-specific level and if caries-related parameters such as depth, stage and activity had been taken into consideration.

## Conclusions

Unlike the saliva samples and the chair-side method, interproximal pooled plaque samples analysed using the “checkerboard DNA-DNA hybridization technique” did not reveal any significant relations between the bacterial counts and the caries experience.

## Competing interests

The authors report no conflicts of interest.

## Authors’ contributions

AM participated in the study design, carried out data collection and sampling, ran the “Checkerboard DNA-DNA Hybridization” analysis, performed statistical analysis and was responsible for the manuscript writing. AC participated in the study design and supervised the manuscript writing. GC assisted in the statistical analysis and helped to draft the manuscript. PL participated in the study design and supervised the manuscript writing. All authors read and approved the final manuscript.

## Pre-publication history

The pre-publication history for this paper can be accessed here:

http://www.biomedcentral.com/1472-6831/13/5/prepub
